# Clinicomicrobiological spectrum of infective endocarditis - from a tertiary care centre in south India

**Published:** 2017-10

**Authors:** Kanne Padmaja, Sukanya Sudhaharan, Lakshmi Vemu, Oruganti Sai Satish, Padmasri Chavali, Mamidi Neeraja

**Affiliations:** 1 Department of Microbiology, Nizam’s Institute of Medical Sciences, Hyderabad, Telangana, India; 2 Department of Cardiology, Nizam’s Institute of Medical Sciences, Hyderabad, Telangana, India

**Keywords:** Native valve endocarditis, Prosthetic valveendocarditis, Congestive heart failure

## Abstract

**Background and Objectives:**

Infective endocarditis (IE) is a microbial infection of the endothelial surface of the cardiac-valves. Rapid diagnosis, effective treatment and prompt recognition of complications are essential, in order to improve the outcome. We retrospectively reviewed and determined the clinical characteristics, microbiological profile and management strategies of IE cases, changing microbial spectrum of pathogens and outcome in Native Valve Endocarditis (NVE) and Prosthetic Valve Endocarditis (PVE) cases.

**Materials and Methods:**

We retrospectively reviewed the medical records of 191 patients, clinically diagnosed with IE, based on modified Dukes criteria, from January 2011 to December 2016. Blood cultures received from all these patients were processed, using BacT/Alert system (bioMerieux, Marcy l’Etoile, France).

**Results:**

Sixty eight (68/191) cases were positive for bacterial pathogens. Twenty four (24/191) cases had PVE and 167/191 had NVE. Nineteen cases (19/24, 79.1%) were PVE positive and forty nine (49/167, 29.3%) were NVE positive. Culture negative endocarditis cases were 123/191 (64.39%). The most common pathogen isolated from NVE cases, in our study was *Streptococcus mitis*, followed by methicillin-resistant coagulase negative staphylococcus (MRCONS) in PVE. The NVE were treated intravenously with a combination of a β-lactam or glycopeptide with an aminoglycoside, for prolonged period of 4–6 weeks, with a successful outcome. The PVE cases were treated with the appropriate antibiotics as per the antibiotic susceptibility report.

**Conclusion:**

The high morbidity and mortality rates are associated with IE and hence accurate identification of aetiological agents and appropriate antimicrobial therapy is required.

## INTRODUCTION

Infective endocarditis (IE) is the infection of lining of the heart or the valves, often affecting the muscles of the heart. It is a life threatening infection with high morbidity and mortality, in case if not aggressively treated with antibiotics or surgery (1). Despite the availability of improved diagnostic and therapeutic facilities, it remains a serious cardiac problem (2). The reported incidence of IE is between 1.7 and 6.2 per 100,000 cases per year, and it has been on the increase and been changing in recent years (3). Overall mortality remains increased, ranging from 21–50%, over the past three decades with an operative mortality of 5–30%, despite recent advances in diagnosis, medical and surgical management of patients with IE (4). The epidemiology, clinical and microbiologic spectrum of IE is different in Indian population, compared to the west and usually depends on the type of endocarditis (native valve or prosthetic) (5). In most developed countries, NVE accounts for 84.5% of cases and PVE accounts for 7–25% of cases of IE (5). The changing spectrum of IE was described through several data available from the developed countries (4). Chronic rheumatic heart disease was found to be the leading cause of chronic valvular disease, comprised of 46% of all cases. Common organisms causing IE include streptococci, staphylococci, enterococci and fastidious Gram-negative coccobacilli. Other rare causes are mycobacteria, rickettsia, chlamydia and fungi (1). *Staphylococcus aureus* remained the most common cause of bacterial endocarditis in India (6–8).

Inspite of high incidence of rheumatic heart disease and unrepaired congenital heart defects in India and other developing countries, there are relatively limited data on the profile and outcome of IE (4). We retrospectively reviewed and determined the clinical characteristics, microbiological profile and management strategies of IE cases, and also focussed on the changing microbial spectrum of pathogens in NVE and PVE cases with outcome.

## MATERIALS AND METHODS

This retrospective study was conducted between Jan 2011 to Dec 2016 (for a period of five years). Data collection was based on the systematic review of all-patient charts from medical records department in order to determine the clinical and microbiological pectrum, 191 patients records, definitely diagnosed with IE, based on modified Dukes criteria were re-viewed and included. Demographic details such as age, sex, clinical findings, and antibiotic usage were analysed from these cases. Three sets of blood cultures, BacT/Alert FNaerobic and SN aerobic bottles per set (bioMerieux, Marcy l’Etoile, France), were submitted to the microbiology lab, within 24 hours of admission and immediately loaded into BACT/ALERT 3D system. Gram staining was performed on all the positive flagged bottles and further sub-cultured on Blood (COS bioMerieux, Marcy l’Etoile, France) and Chrome agar (COS bioMerieux, Marcy l’Etoile, France) plates and incubated at 37°C for 24h. The Vitek 2 (bioMérieux, Marcy l’Etoile, France) GN cards (ID GN panel) were used, for accurate identification of Gram-negative pathogens and Vitek 2 AST Cards (N281 panel), for antimicrobial susceptibility testing.

The Vitek 2 GP cards (ID GP) were used, for accurate identification of Gram-positive pathogens, and Vitek 2 AST cards for antimicrobial susceptibility testing.

## RESULTS

The age range of the patients were 17–80 years, with a male dominance (male: female-1.2:1). The mean age of the IE cases was 30 years, ranging between 21 and 84 years. Majority were in the age group of 41–50 years (Table 1). Among 191 cases, 24/191 cases had PVE, 167/191 had NVE. Blood cultures were positive in 68 patients, out of which 19/24 (79.1%) are PVE positive and 49/167 (29.3%) are NVE positive. Culture negative endocarditis was observed in 123/191 (64.39%) cases (Table 2).

**Table 1.** Age distribution of patients (Total n=191)

**Age (year)**	**Number of patients**
0–20	2
21–30	15
31–40	45
41–50	90
51–60	32
61–70	4
71–80	3

**Table 2.** Distribution of NVE & PVE cases (Total n = 191)

**Type of Endocarditis**	**N (%)**	**Culture positive N (%)**	**Culture negative N (%)**
NVE	167 (87.43%)	49 (29.3%)	118
PVE	24 (12.5%)	19 (79.16%)	5

128/191 of our patients (67%) had Rheumatic heart disease (RHD), as an underlying cardiac condition, followed by congenital heart disease 10/191 (5.2%), ventricular septal defect in 10/191 (5.2%), atrial septal defect 10/191 (5.2%), mitral valve prolapse in 9/121 (4.7%) and prosthetic valve disease in 24/191 patients (12.5%) (Table 3).

**Table 3.** Underlying cardiac conditions among IE patients (n=191)

**Variables**	**Number**	**Percentage (%)**
Rheumatic Heart disease	128	67.0
Prosthetic valves	24	12.5
Congenital heart disease	10	5.2
Ventricular septal defect	10	5.2
Atrial septal defect	10	5.2

49/167 cases were culture positive for NVE. The predominant organism isolated include *Streptococcus mitis* (n=20), followed by *Streptococcus sanguinis* (n=13). The complete distribution of causative microorganisms is shown in (Table 4).

**Table 4.** Microbial spectrum of NVE & PVE cases

**Microbial spectrum of NVE cases (n=49)**	**Number of isolates N (%)**	**Microbial spectrum of PVE cases (n=19)**	**Number of isolates N (%)**
*Streptococcus mitis*	20 (40.8)	Methicillin Resistant *Staphylococcus aureus* (MRSA)	4 (21.05)
*Streptococcus sanguinis*	13 (26.5)	Methicillin susceptible *Staphylococcus aureus* (MSSA)	2 (10.52)
*Streptococcus pyogenes*	3 (6.12)	Methicillin Resistant Coagulase negative *Staphylococcus* (MRCONS)	7 (36.84)
*Streptococcus pneumoniae*	1 (2.04)	*Klebsiella pneumoniae*	3 (15.78)
*Granulicatella adiacens*	1 (2.04)	*Achromobacter denitrificans*	3 (15.78)
*Abiotrophia defectiva*	1 (2.04)	*Burkholderia cepacia*	3 (15.78)
*Gemella morbillorum*	2 (4.08)		
Methicillin Resistant *Staphylococcus aureus* (MRSA)	1 (2.04)		
Methicillin susceptible *Staphylococcus aureus* (MSSA)	2 (4.08)		
*Brucella melitensis*	2 (4.08)		
*Brevundimonas diminuta*	1 (2.04)		
*Stenotrophomonas maltophilia*	1 (2.04)		
*Corynebacterium diphtheriae*	1 (2.04)		

Culture positivity was observed in 19/24 cases of PVE. MRCONS was the predominant organism isolated. The complete distribution of causative microorganisms is shown in Table 4.

Fever was the most common clinical feature, observed in majority of cases (n=54, 28.2%), followed by sudden onset of breathlessness (n=24, 12.5%) (Table 5).

**Table 5.** Clinical symptoms in IE patients (Total n=191)

**Clinical Symptoms**	**Number of patients**	**Percentage (%)**
Fever	54	28.2
Sudden onset of breathlessness (SOB)	24	12.56
Cough	20	10.47
Heart murmur	24	12.56
Chest pain	20	10.47
Dyspnoea	18	9.42
Tachycardia	6	3.14
Tachypnoea	5	2.61

A transthoracic echocardiography (TTE) was performed for all the patients. Vegetations were present in 120/191 (62.8%) patients and the most common site of vegetation was mitral valve, which was detected in 65/120 patients (54.16%). The other findings was shown in (Table 6).

**Table 6.** Sites of vegetations in IE patients (Total n=120)

**Site**	**N**	**Percentage (%)**
Mitral valve	65	54.16
Aortic valve	20	16.6
Tricuspid valve	20	16.6
Pulmonary valve	15	12.5

## DISCUSSION

IE is the infection of the endothelial surface of the heart (2). Despite advances in medical, surgical and critical care interventions, the incidence of IE has not been changed over the past two decades (2, 9). Active IE is lethal, if not aggressively treated with antibiotics, combined or not associated with surgery (1).

In developed countries, most of the studies on IE, had been seen in elderly and have demonstrated a gradual increase in the mean age of IE patients (1). But majority of our patients were younger and our data are consistent with previously published studies from developing countries (2), which may be due to the higher rate of chronic rheumatic heart disease (CRHD). In the European heart survey, it was observed that bacterial endocarditis accounted for 26% and most of the affected patients were elderly group with an age of more than 70 years but in the Indian populations, it is still common in younger age group, as similar to our study (10).

In our study, CRHD was the most common predisposing factor similar to other studies (9, 11, 12). In a recent study from Turkey, CRHD was found to be the leading cause of chronic valvular disease, which comprised of 46% of all cases (13).

Our study, also confirmed many clinical features of endocarditis, occurring in other parts of the world. Fever was the most common clinical feature (28.2 %), observed among our patients with similar percentage reported by other study (14). The lack of fever should not exclude the diagnosis of IE, in a patient with suggestive clinical features.

Diagnosis of IE by TTE/TEE (Trans oesophageal echocardiography) is a non-invasive and available method of choice, in order to evaluate the presence of vegetations (14). Thus, TEE is highly sensitive for detecting IE. In our study, vegetations were detected in 62.8% of patients by TTE only. Mitral valve was the most common valve affected in our study (54.16%), similar to other studies (4, 15).

We have observed in our study, that NVE is common (87.4%), which is in close agreement with previous studies (9, 16), where as Senthil et al. (4) have observed, in 95.7% cases.

PVE accounted for 7–25% of cases of IE, in most developed countries (9). In developing countries like India, Senthil et al. (4) observed PVE in 4.3% of cases and Mario et al. (9) in 30% of cases. PVE depends on the time of onset of endocarditis, following the valve replacement. Approximately 40–60% of early onset PVE is caused by both Gram-positive cocci and Gram-negative bacteria. In our study the frequency of IE involving prosthetic valves was around 12.5% and mostly caused by Gram-positive cocci (9).

IE is caused by a wide variety of pathogens. Most commonly staphylococcus spp. and streptococcus spp. are identified, but new species are constantly being discovered (2). Common species causing IE include streptococci, staphylococci, enterococci and fastidious Gram-negative coccobacilli. Other rare causes are mycobacteria, rickettsia, chlamydia and fungi (2).

*Streptococcus viridans* is responsible for 30 to 65% of NVE in India, thus *Staphylococcus* incidence is on the raise. Our study also showed that *S. viridans* has overcome *S. aureus*, as the most common cause of IE particularly in NVE. The previous Indian study by Garg et al. has shown that streptococci are the most common isolate (17), which was consistent with our observation.

Murdoch et al. (15) observed that *S. aureus* is the most common cause of IE worldwide, due to the presence of risk factors such as intravenous drug use and invasive devices. The increasing emergence of *Staphylococcus* in west, is accounted by an increasing geriatric population, rising drug abuse, increasing nosocomial infections and use of prosthetic devices. *S. aureus* was the most common causative agent identified in North America, Turkey and Saudi Arabia (10), but in our study we isolated 6.1% of *S. aureus* isolates.

Coagulase-negative staphylococci (CONS) cause about 6% of NVE, but rarely acute IE. In our study, we observed that CONS was isolated in most cases of PVE (36.8%).

The high yield of CONS is usually surprising, because isolation of these organisms suggest the skin contamination. But in the presence of prosthetic valve, isolation of this pathogen should be considered significant.

Thus, the incidence of pneumococcal endocarditis is not exactly known, but few clinical studies have estimated the prevalence to be less than 3%. Usually *Streptococcus pneumoniae* is known to cause respiratory tract infections but many recent studies have greatly emphasized on their role, in causing bacterial endocarditis as similar to our study. Because as such pneumococcal bacteremia is usually the consequence of lung infection.

A case of PVE caused by *Achromobacter denitrificans* in a 17 years old patient, with known congenital heart disease and aortic stenosis who had undergone surgical replacement of mitral valve has been examined in our study. The most common manifestation of infection documented with this organism is bacteremia, with a mortality rate of more than 50%. PVE, usually develops between 4–6 months of valve replacement due to *Achromobacter xylosoxidans* subsp denitrificans (18), as also seen in our case (19). It is probable that our patient acquired the valve infection, either intra-operatively or postoperatively.

Thus some unusual IE pathogens such as NVS, *Gemella morbillorum* and *Brucella melitensis* that are slow growing and difficult to be isolated from blood cultures, were isolated from our cases because of availability of commercial liquid media that supports the growth of the pathogens and advanced automated systems.

NVS are the etiological agents of IE in 5%–6% of cases (20). We isolated *G. adiacens* from a NVE patient. Giuliano et al. (21). also reported NVS in a patient with IE caused by *G. adiacens*, similar to our case (22). In our case, the route of entry of NVS into the bloodstream was not known and these organisms are assumed to have originated from the oral cavity, as the patient had bad oral hygiene, for which she had undergone dental manipulations several times, before the episode of IE.

*Gemella* species are small Gram-positive cocci, a rare cause of endocarditis that have been increasingly reported since 1982. Taimur et al. reported twenty-four cases of *Gemella* endocarditis in the literature, up to 2010 (23). We reported only 2 cases of NVE caused by *G. morbillorum* in our study.

Brucella IE is an uncommon, but life threatening complication of brucellosis, it is observed in less than 2% of the brucellosis cases. The aortic valve is the most commonly affected cardiac valve (24). We also had a similar case of Brucella endocarditis (BE), where aortic valve was affected and *B. melitensis*, isolated from aortic valve tissue and also blood cultures. Mustafa et al. reported 31 cases of native BE (25).

Thus, bacterial isolation from valvular tissue is not always possible; healed endocarditis, broad spectrum perioperative antibiotic therapy, systemic hypothermia, haemodilution and cold cardioplegia reduce the possibility of pathogen isolation from the infected tissue (1). In our study, *Brucella* was isolated from aortic valve tissue from a case of BE.

Corynebacteria have been shown to be responsible for 0.2 to 0.4% of IE cases, in native valve (26). In our study we reported a case of NVE in a 9 years old child with Tetrology of Fallot (TOF) with sub-aortic ventricular septal defect (VSD) caused by non-toxigenic (NT) *C. diphtheriae* biotype mitis. But due to cardiac arrest patient was succumbed to death, though aortic valve replacement was planned (27). The NT *C. diphtheria* probably entered the body, either subsequent to skin and/or throat colonization or a percutaneous trauma.

*Stenotrophomonas maltophilia* is a nosocomial pathogen resistant to multiple antibiotics and is a rare cause of endocarditis and carries high mortality and morbidity (28). In our study, *S. maltophilia* was identified as a cause of active IE from a native aortic valve of patient.

Positive blood culture is a major diagnostic criterion for IE. Blood culture is often negative in Indian population, compared to western due to prior antimicrobial therapy. Failure to culture the organism may result from inadequate antimicrobial technique, infection with highly fastidious pathogen or most importantly, because of the administration of antimicrobial agent before blood cultures are performed (2).

Most of our patients had previous use of antibiotics prior to hospital admission, which was the main reason for high number of culture negative cases. This is consistent with previous reports (17), in which the number of patients with culture negative endocarditis, received antibiotics in the 2 weeks prior to diagnosis, was found to be as high as 64.3% in our cases. Senthil et al. (4) reported 76.7% of CNE, which was still more higher, compared to our study. In Syed Mohammed study, 46.7% of patients were found to be culture negative (14).

The total surgeries done for IE in our study was 32 (35.16%), the most common was mitral valve replacement 22 (11.5%), followed by aortic valve replacement 8 (4.18%) and VSD closure in 2 (1.04%) patients. All patients with streptococcal IE, should be treated for at least 2 weeks in hospital and observed for cardiac and non-cardiac complications.

IE caused by methicillin-resistant *S. aureus* (MRSA), is a therapeutic challenge as most strains are also resistant to most aminoglycosides. If the clinical course is complicated, treatment should be as for PVE.

Coagulase-negative species (CONS), causing PVE within the first year after valve replacement are usually methicillin-resistant. Therapy of choice is a combination of vancomycin and rifampicin for at least 6 weeks with the addition of gentamicin for the initial two weeks (28).

Enterococci are generally resistant to a wide range of antimicrobial agents including aminoglycosides (MIC for gentamicin 4–64 mg/l). Duration of treatment should be at least 4 weeks for the combination and at least 6 weeks in complicated cases, for patients having symptoms for more than 3 months, and for patients with PVE (28).

Aminoglycosides are often used in combination with a cell wall-active agent (β-lactam or vancomycin), for synergy in the treatment of IE caused by staphylococci, streptococci and enterococci. Cell wall-active agents increase aminoglycoside entry into bacteria, and therefore synergy requires dosing in close temporal proximity to one another (29).

All the NVE cases, in our study were treated with a combination of a β-lactam or glycopeptide with an aminoglycoside mostly ceftriaxone and gentamicin, intravenously for prolonged period of 4–6 weeks, with a successful outcome.

All the PVE cases, in our study were treated with vancomycin, in case of Gram-positive pathogens and meropenem was used against Gram-negative pathogens.

Jain et al. (12) reported the mortality rate in patients who underwent surgery was 6 (8.3%) and Fahriye et al. (10) showed mortality of 30% and both studies observed that CHF was the most common complication leading to death. Loupa et al. reported an overall mortality rate of 16% (12). Whereas, mortality rate among our patients with IE was 13 (40.6%), which was comparatively high, compared with other study. Main causes of death in our study were sepsis in 10 (31.25%), cerebral embolism in 2 (6.25%) and cardiac arrest in 1 patient (3.12%).

## CONCLUSION

Despite recent advances, the management of IE remains a serious and challenging problem. Patients with endocarditis need more accurate clinical evaluation, high index of clinical suspicion, early diagnosis for at risk populations, which can prevent disease progression.

The accurate identification of aetiological agents and appropriate antimicrobial therapy are associated with IE. We observed that *Streptococcus mitis* was the most predominant pathogen that led to bacterial endocarditis in our cases. All our cases were promptly treated with conservative management. And the cases which did not respond were posted for surgery with good clinical outcome. Congestive heart failure was the most frequent infective endocarditis complication as well as indication for surgery. In-hospital mortality rate of patients was unexpectedly low.

Results obtained in our study emphasizes that there is an urgent need for implementation of strict infection control measures and practices to reduce the incidence of IE which is most often due to hospital acquired pathogens. Henceforth surveillance studies, should be initiated with the existing global scenario in prevention, diagnosis and management of IE cases.
